# Preoperative Oral Gabapentin in the Management of Postoperative Catheter-Related Bladder Discomfort in Adults: A Systematic Review and Meta-Analysis

**DOI:** 10.3389/fsurg.2021.755497

**Published:** 2021-10-18

**Authors:** Yu-Ting Wang, Chong Xiao, Hong Liu, Xi Fu, Yi-Feng Ren, Feng-Ming You

**Affiliations:** TCM Regulating Metabolic Diseases Key Laboratory of Sichuan Province, Hospital of Chengdu University of Traditional Chinese Medicine, Chengdu, China

**Keywords:** catheter-related bladder discomfort, gabapentin, efficacy, meta-analysis, postoperative complications

## Abstract

**Objective:** To evaluate the efficacy and safety of preoperative oral gabapentin in preventing postoperative Catheter-Related Bladder Discomfort (CRBD) in surgical patients.

**Methods:** Randomized controlled trials in which gabapentin was used for the prevention of CRBD in surgical patients with transurethral catheterization were evaluated. The primary outcome was the incidence of moderate-to-severe CRBD at 0, 1, 2, and 6 h after surgery, and secondary outcomes included the incidence of any grade CRBD, postoperative pain, and adverse events. Pooled risk ratios (RRs) and mean difference (MD), 95% confidence intervals (CIs), and *P* values were estimated using fixed and random effects statistical models. The Grading of Recommendations Assessment, Development, and Evaluation (GRADE) approach was used to rate the levels of certainty for key results.

**Results:** A total of 6 randomized controlled trials involving 679 participants were included in the meta-analysis. Gabapentin significantly reduced the risk of moderate-to-severe CRBD at 0, 1, 2, and 6 h (0 h: RR = 0.19, 95% CI: 0.11 to 0.31, *p* < 0.00001; 1 h: RR = 0.40, 95% CI: 0.25 to 0.66, *p* < 0.001; 2 h: RR = 0.38, 95% CI: 0.26 to 0.56, *p* < 0.00001; 6 h: RR = 0.20, 95% CI: 0.11 to 0.38, *p* < 0.00001). The overall incidence of CRBD at 1 h showed no statistical difference between the two groups (RR = 0.55, 95% CI: 0.30 to 1.00, p = 0.05). The risk of CRBD was significantly reduced in the gabapentin group at 0, 2, and 6 h after surgery (0 h: RR = 0.59, 95% CI: 0.46 to 0.74, *p* < 0.0001; 2 h: RR = 0.62, 95% CI: 0.51 to 0.75, *p* < 0.00001; 6 h: RR = 0.66, 95% CI: 0.52 to 0.83, *p* < 0.001). In addition, gabapentin was associated with low postoperative pain intensity without significant side effects.

**Conclusion:** Preoperative oral gabapentin as an adjunct to surgery is effective in decreasing the risk and severity of CRBD over a short time after surgery, and it can decrease postoperative pain without significant side effects. Overall, the level of certainty was moderate to low.

**Systematic Review Registration:** https://www.crd.york.ac.uk/prospero/#recordDetails, identifier: CRD42021228171.

## Introduction

Catheter-related bladder discomfort (CRBD) secondary to an indwelling urinary catheter is a prevalent complication associated with most surgeries, which is characterized by a burning sensation spreading from the suprapubic area to the penis, urinary frequency and urgency, with or without urge incontinence (1, 2). Approximately 47–90% of patients under general anesthesia suffer from CRBD, which leads to increased postoperative agitation, poor patient satisfaction, prolonged hospitalization, and increased workload for healthcare workers (3, 4). Therefore, aggressive prevention and appropriate treatment for CRBD are necessary.

Despite various agents have been applied to prevent and treat CRBD, there is still no consensus on the best choice of drug used in the symptomatic relief of CRBD (5–7). Gabapentin has been believed to a promising prevention agent (4, 8, 9) and there are several randomized controlled trials (RCTs) which have examined the effectiveness of preoperative oral gabapentin in decreasing the incidence and severity of postoperative CRBD. However, the evidence has not been sufficiently robust to guide clinical decision-making. With a crescendo of voices expressing concern about potential adverse events and net clinical benefit of gabapentin, compelling evidence is required to avoid its abuse and misuse.

The aim of this study was to determine the efficacy and safety of preoperative gabapentin in preventing postoperative CRBD, especially moderate-to-severe CRBD among patients subjected to surgery with indwelling urinary catheters by performing a systematic review and meta-analysis.

## Methods

### Search Strategy

The present systematic review was performed according to the Preferred Reporting Items for Systematic Reviews and Meta-Analysis (PRISMA) guidelines using the PICOS framework (10). The research had been registered prospectively on PROSPERO (CRD42021228171). Two reviewers (YTW and CX) independently searched PubMed, Cochrane Library, EMBASE, Web of Science, China National Knowledge Infrastructure (CNKI), Wanfang, Weipu, and Google Scholar databases from the dates of their inception to July 2021 (the search strategies are shown in Supplementary Material). Furthermore, references of retrieved articles and relevant review papers were searched manually. The inclusion criteria were as follows: (1) population: studies in which the participants were adult (age >18 years) human patients undergoing surgery with indwelling urinary catheters; (2) intervention: preoperative oral administration of gabapentin ≥30 min before the surgical procedure; (3) comparison: placebo or no treatment; (4) predefined outcomes: incidence of postoperative CRBD at different time points, and (5) design: RCTs published without language restrictions and full-text versions. The exclusion criteria were as follows: (1) case report, conference abstract, or review article; (2) unpublished trials.

### Data Extraction

Two investigators (HL and XF) independently checked the eligibility of the published studies, extracted data, and assessed the risk of bias. Disagreements were resolved by discussion among reviewers. The following demographic and clinical data were extracted to an Excel spreadsheet: first author name, publication year, sample sizes, age, gender, detailed intervention methods for each group, type of surgery, method of anesthesia administration, and outcome parameters.

Standard criteria for bladder discomfortable were employed, which was divided into four grades: no, mild, moderate and severe discomfort (the precise grading standard was given in the Supplementary Table 1). The primary outcome was the incidence of moderate-to-severe CRBD at 0, 1, 2 and 6 h after surgery. The secondary outcomes included the overall incidence of CRBD, postoperative pain scores, and adverse events. Risk of bias was assessed independently by two researchers using the Cochrane Collaboration's tool across six domains (selection, performance, detection, attrition, reporting, and other), and was classified as high, low, or unclear (11). The levels of certainty for key results were evaluated based on the guidelines of the Grading of Recommendations Assessment, Development, and Evaluation (GRADE) system.

### Statistical Analysis

Meta-analyses were conducted using Review Manager (RevMan version 5.3: The Nordic Cochrane Center, The Cochrane Collaboration, Copenhagen, Denmark, 2014). We used risk ratio (RR) with 95% confidence interval (CI) for dichotomous data and standard mean difference (SMD) or mean difference (MD) with 95% CI for continuous data (12). Heterogeneity was calculated using I^2^ statistic; for significant heterogeneity (I^2^ ≥ 50%), random rather than fixed-effects models were used. We performed subgroup and sensitivity analyses to investigate possible sources of heterogeneity when heterogeneity of primary outcome was significant. In addition, subgroup analysis was only performed when at least two trials in a specific subgroup. A *P* value < 0.05 was considered statistically significant.

## Results

### Selection and Characteristics of Studies

**Figure 1:**
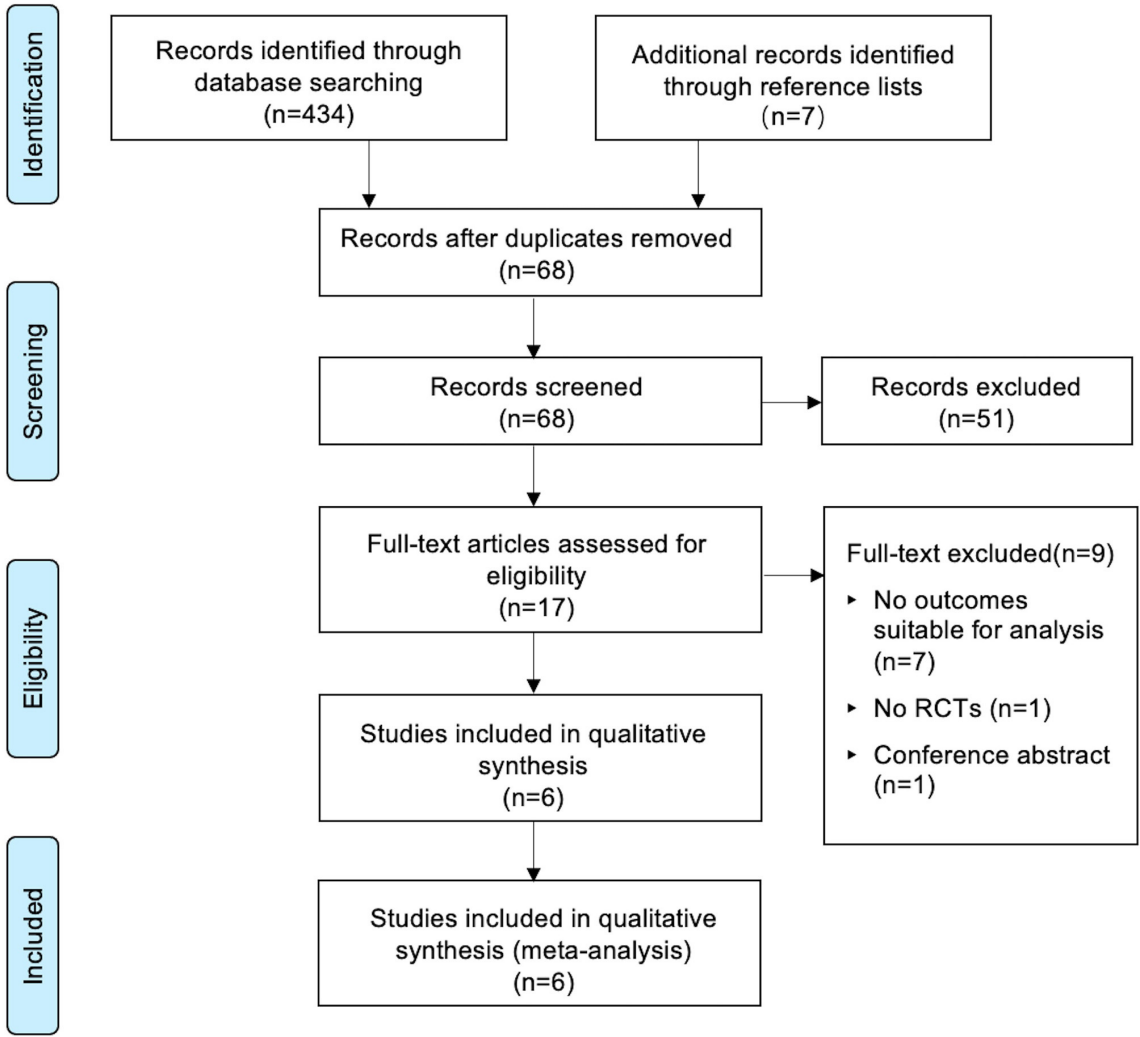
Flowchart of the study selection.

The PRISMA checklist shown in Supplementary Checklist, and the PRISMA flow chart for the primary literature selection process are shown in Figure 1. A total of 196 studies were identified from an initial search of the databases and other sources. Among them, 6 RCTs with 679 patients satisfied the inclusion criteria and were used for the systematic review (13–18). The articles were published from 2007 to 2020 and the sample sizes ranged between 40 and 181. All patients had transurethral catheterization under general anesthesia with an exception of one study in which spinal anesthesia was used (14). All included studies evaluated the effect of preoperative oral gabapentin on postoperative CRBD at a dose of 600 mg, administered 1 or 2 h before surgery. The comparisons were as follows: five comparisons of placebo controls to gabapentin experimental arms (13, 19) and one blank control (18). Two studies investigated the role of gabapentin in percutaneous nephrolithotomy (13, 15), one study involved transurethral resection of bladder tumor (14), participants in one study underwent transurethral resection of the prostate (16), one study involved abdominal hysterectomy (18), and participants in one study received flexible ureteroscopes (17). In all studies, catheterization was done by using a 16-Fr Foley catheter, except one (16) without specifying the size of catheter and one (17) using a 16-Fr Foley catheter for man while a 12-Fr Foley catheter for woman. No baseline difference between groups was observed in all included studies. The characteristics of the included RCTs are summarized in Table 1.

**Table 1:** Characteristics of included studies.

**Study**	**Type of surgery**	**Timing of intervention**	**Intervention in each arm**		**No ineach arm**	**Sex: M/F**	**Age (yr)**	**Duration of surgery (min)**	**Inclusion criteria**	**Exclusion criteria**	**Foley cath.size**	**Anesthesia**	**Timing of assessment**
**Agarwal et al**. **(****13****)**	PCNL	1 h before the induction of anesthesia	Interv-ention	Gabapentin 600 mg p.o.	54	42/12	37.3 (9.6)	148 (28)	• ASA I-II	• Age > 60 yrs • Chronic opioid use • Bladder outflow obstruction • TURP for BPH	16 Fr	GA	0, 1, 2, 6 h after arrival in the PACU
			Control	Placebo p.o.	54	47/7	40.8 (13.9)	155 (34)		• Overactive bladder • Neurogenic bladder • DM • Parkinson's disease • ESRD (UO <500 mL/24 h)			
**Bala et al**. **(****14****)**	TURBT	1 h beforethe	Interv-ention 1	Gabapentin 600 mg p.o.	33	28/5	52.3 (7.2)	58.4 (18.7)	• 20-60 yr, • ASA I-II	• Overactive bladder • Neurogenic bladder	16 Fr	SA	1, 2, 4, 6, 12, 24 h after
		administrati-on of spinal anesthesia	Interv-ention 2	Gabapentin 1200 mg p.o.	34	25/9	52.4 (6)	65.9(8.3)		• Impaired renal function • Chronic use of opioids or sedatives • Antacid uptake in the past 48 h			arrival in the PACU
			Control	Placebo p.o.	33	26/7	52 (6.3)	65.8(8.2)		• Hypersensitivity to amide local anesthetics or gabapentin			
**Maghso-udi et al**. **(****15****)**	PCNL	1 h beforesurgery	Interv-ention 1	Gabapentin 600 mg p.o.	50	NA	39.4 (10.0)	98.3(26.8)	• 18-60 yr, • ASA I	• Drug or alcohol abuse • Allergic reactions to gabapentin, tolterodine or • Narcotics • Painful circumstances which can affect pain	16 Fr	GA	1, 3, 12, 24 h after surgery
			Interv-ention 2	Tolterodine 2 mg p.o.	50		44.4 (9.7)	97.9(19.7)		assessment including lower urinary tract symptoms • Medical or psychologic circumstances which can affect the patients' communication or tolerance • Analgesic use within 12 h before surgery • Urethral pathologies necessitating intervention			
			Control	Vitamin C 250mg p.o.	70		44.1 (12.2)	105.9(23.4)		• or causing difficulty in passage of urethral catheter • Change in anesthesia protocol during the operation			
**Wang et al**. **(****16****)**	TUPR	2 h prior to TUPR	Interv-ention 1	Gabapentin 600 mg p.o.	90	Allmale	66.9 (9.5)	56.8(12.3)	• ASA I-II	• Allergy to the study medication • Renal or hepatic insufficiency	NA	GA	2, 4, 8, 16, 24, 36, 48 h after tracheal extubation
			Control	Placebo p.o.	91		68.1 (8.8)	59.7(19.7)		• Receiving analgesics within 48 hours prior to surgery • Chronic pain, drug or alcohol abuse • Psychiatric disorder			
**Yang et al**. **(****18****)**	AH	1 h before the induction of anesthesia	Interv-ention 1	Gabapentin 600 mg p.o.	40	Allfemale	44.5 (14.1)	128.0(10.4)	• Weight: 45~100 kg • ASA I-II	• Catheterization process not smooth • Overactive bladder • ESRD	16 Fr	GA	0, 1, 2, 6 h after arrival in the PACU
			Control	None	40		45.0 (9.6)	125.0(11.7)		• Cardiovascular disease • Liver disease • Morbid obesity • Central nervous system dysfunction • Psychiatric disorder • Chronic pain and drug abuse			
**Cheng et al**. **(****17****)**	FU	2 h before surgery	Interv-ention	Gabapentin 600 mg p.o.	20	12/8	47.31 (9.97)	61.13(29.73)	• ASA I-II	• Allergy to the Gabapentin • Epileptics	16 Fr for male; 12 Fr for female	GA	0, 1, 2, 6 h after surgery
			Control	Placebo p.o.	20	11/9	46.35 (11.01)	59.24(30.11)		• Use history of gabapentin • Renal or hepatic insufficiency			

*Values are shown as mean (standard deviation) or median (interquartile range) or number. PCNL, percutaneous nephrolithotomy; ASA, American Society of Anesthesiologists; TURP, transurethral resection of the prostate; BPH, benign prostatic hyperplasia; DM, diabetic mellitus; ESRD, End-stage renal disease; GA, general anesthesia; PACU, post-anesthesia care unit; TURBT, transurethral resection of bladder tumor; SA, spinal anesthesia; NA, not available; AH, abdominal hysterectomy; FU, flexible ureteroscope*.

### Risk of Bias Assessment

**Figure 2:**
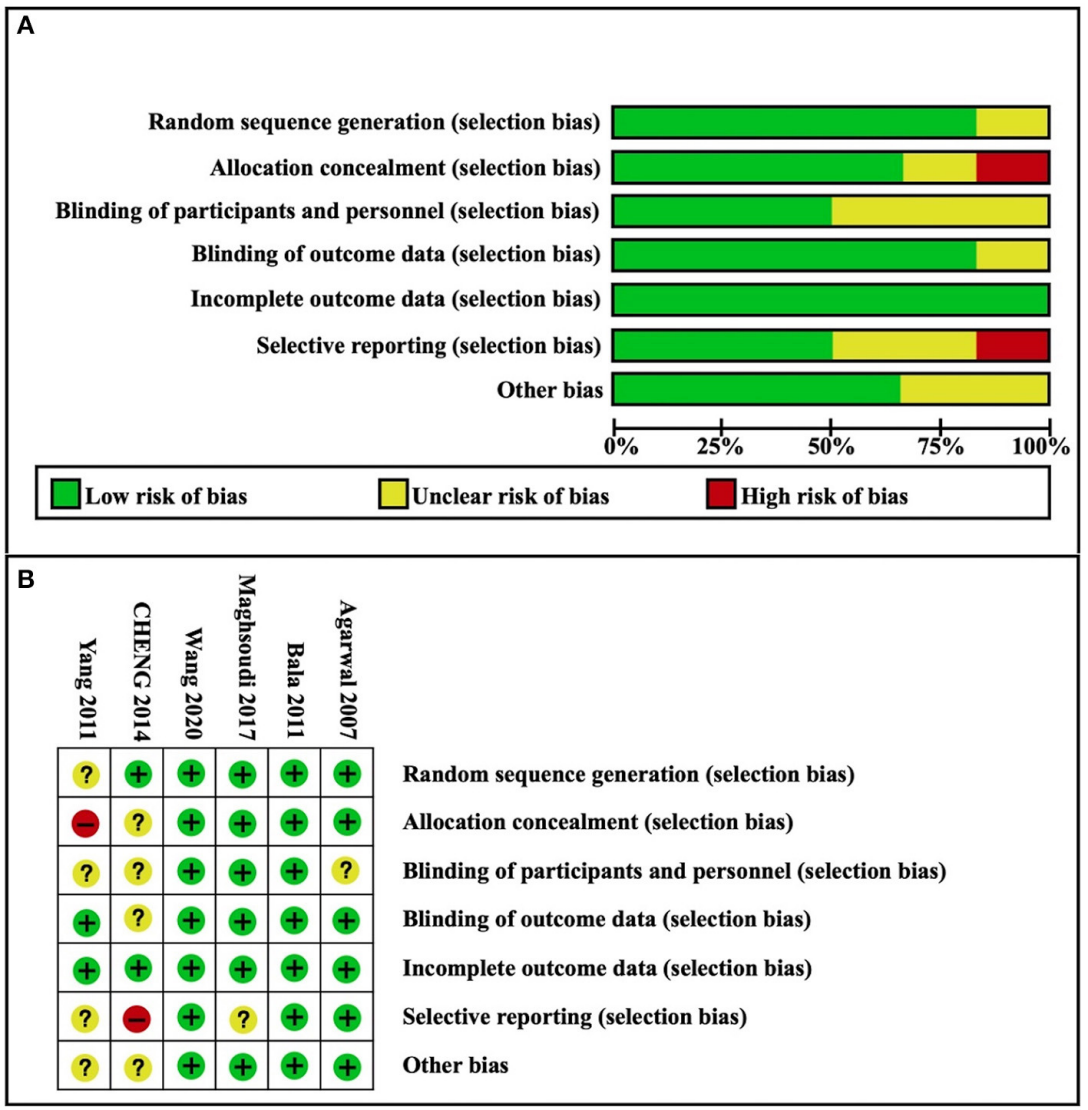
Risk of bias. **(A)** Risk of bias graph; **(B)** Risk of bias summary. Green = low risk of bias; yellow = unclear risk of bias; red = high risk of bias.

Based on the assessment conducted using the Cochrane Collaboration's tool, most of the studies had a “low risk” or an “unclear risk”. A summary of judgments made by reviewers for each risk of bias item for each included study is presented in Figure 2. The levels of certainty for key results are summarized in Table 2.

**Table 2:** The GRADE level of certainty for key results.

**Outcomes**	**No of studies**	**No of patients**	**Quality assessment**					**Effect (95% CI)**	**Quality**	**Importance**
			**Risk of bias**	**Inconsistency**	**Indirectness**	**Imprecision**	**Other considerations**			
Moderate-to-severe postoperative CRBD at 0h	3	228	Serious ^a^	No serious	No serious	No serious	No serious	**RR 0.19** (0.11 to 0.31)	⊕⊕⊕○ MODERATE	Critical
Moderate-to-severe postoperative CRBD at 1h	5	414	Serious ^a^	No serious	Serious ^b^	No serious	No serious	**RR 0.40** (0.25 to 0.66)	⊕⊕○○ LOW	Critical
Moderate-to-severe postoperative CRBD at 2h	4	294	Serious ^a^	No serious	No serious	No serious	No serious	**RR 0.38** (0.26 to 0.56)	⊕⊕⊕○ MODERATE	Critical
Moderate-to-severe postoperative CRBD at 6h	4	294	Serious ^a^	No serious	No serious	No serious	No serious	**RR 0.20** (0.11 to 0.38)	⊕⊕⊕○ MODERATE	Critical
Any severity postoperative CRBD at 0h	2	188	Serious ^a^	No serious	No serious	Serious ^c^	No serious	**RR 0.59** (0.46 to 0.74)	⊕⊕○○ LOW	Critical
Any severity postoperative CRBD at 1h	3	254	Serious ^a^	No serious	Serious ^b^	No serious	No serious	**RR 0.55** (0.30 to 1.00)	⊕⊕○○ LOW	Critical
Any severity postoperative CRBD at 2h	3	254	Serious ^a^	No serious	No serious	No serious	No serious	**RR 0.62** (0.51 to 0.75)	⊕⊕⊕○ MODERATE	Critical
Any severity postoperative CRBD at 6h	3	254	Serious ^a^	No serious	No serious	No serious	No serious	**RR 0.68** (0.55 to 0.84)	⊕⊕⊕○ MODERATE	Critical
Postoperative pain scores at 0h	2	188	Serious ^a^	No serious	No serious	Serious ^c^	No serious	**MD−1.40**(-1.81 to−0.98)	⊕⊕○○ LOW	Critical
Postoperative pain scores at 1h	3	308	Serious ^a^	No serious	Serious ^b^	No serious	No serious	**MD−2.09**(-3.39 to−0.78)	⊕⊕○○ LOW	Critical
Postoperative pain scores at 2h	3	369	Serious ^a^	No serious	Serious ^b^	No serious	No serious	**MD−0.79**(-1.34 to−0.25)	⊕⊕○○ LOW	Critical
Postoperative pain scores at 6h	2	188	Serious ^a^	No serious	Serious ^b^	Serious ^c^	No serious	**MD−1.05**(-1.95 to−0.15)	⊕⊕○○ LOW	Critical
Postoperative nausea and vomiting	3	287	Serious ^a^	No serious	No serious	No serious	No serious	**RR 0.63** (0.28 to 1.45)	⊕⊕⊕○ MODERATE	Critical
Postoperative dizziness	3	301	Serious ^a^	No serious	No serious	No serious	No serious	**RR 0.78** (0.53 to 1.17)	⊕⊕⊕○ MODERATE	Critical

*CI, confidence intervals; RR, risk ratio; MD, mean difference*.

a *Quality was rated down for one or two included studies with a high risk of bias*.

b *Quality was rated down because I^2^ > 50%*.

c *Quality was rated down due to total patient size is less than 200*.

### Effects of Intervention

#### Moderate-to-Severe Postoperative CRBD

**Figure 3:**
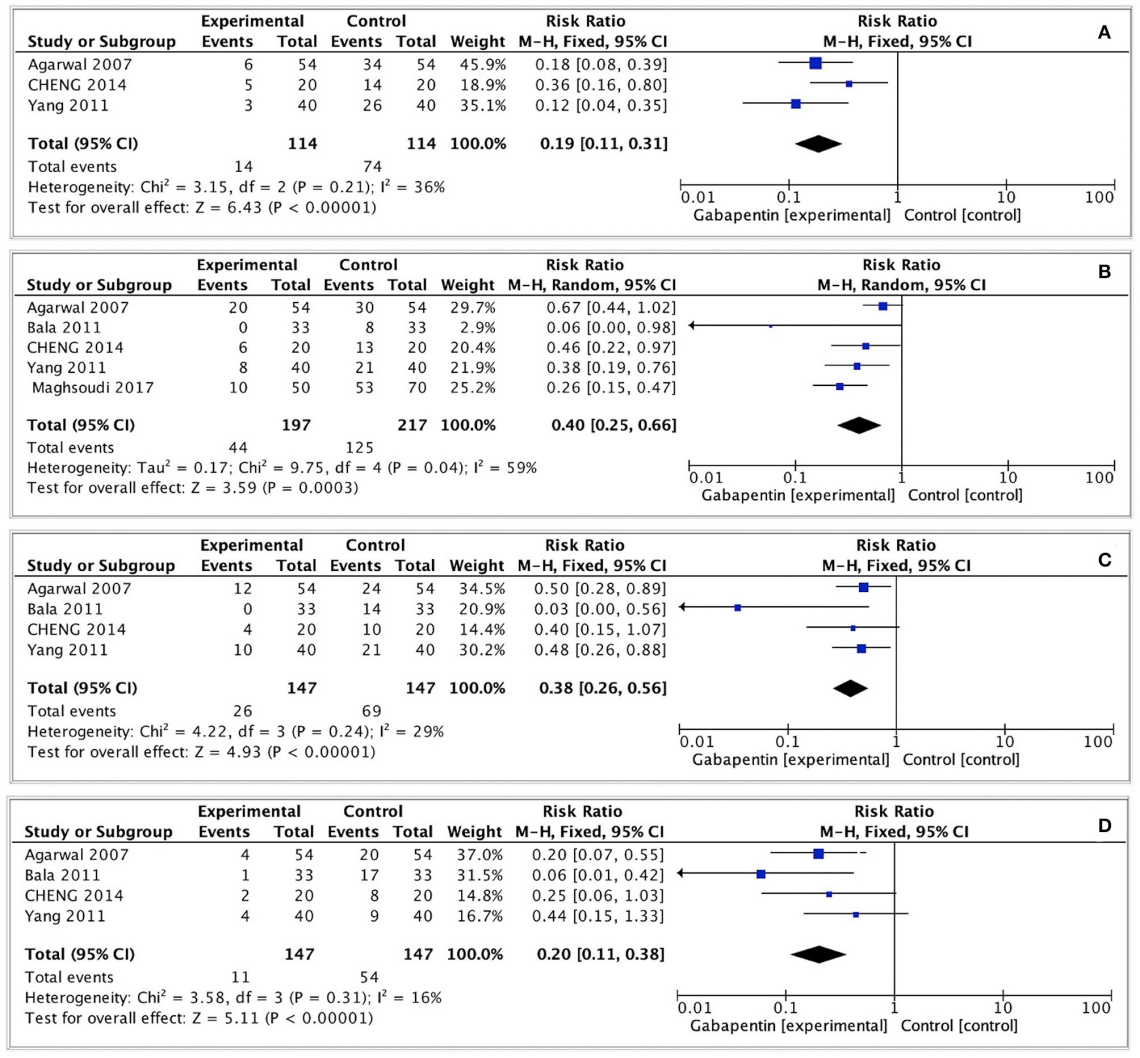
Forest plot of moderate-to-severe postoperative CRBD. **(A)** Moderate-to-severe postoperative CRBD at 0h after surgery; **(B)** Moderate-to-severe postoperative CRBD at 1h after surgery; **(C)** Moderate-to-severe postoperative CRBD at 2h after surgery; **(D)** Moderate-to-severe postoperative CRBD at 6h after surgery.

The incidence of moderate-to-severe CRBD was assessed at 0, 1, 2, and 6 h after surgery. A fixed effects model was used to estimate pooled effect size because no significant heterogeneity was observed among studies at 0, 2, and 6 h (I^2^ = 36%; 29%; 16%). A random effects model was used to calculate pooled effect size at 1 h for significant heterogeneity among studies (I^2^ = 59%). The incidence of moderate-to-severe CRBD reduced significantly at each time point (0 h: RR = 0.19, 95% CI: 0.11 to 0.31, *p* < 0.00001, GRADE = moderate; 1 h: RR = 0.40, 95% CI: 0.25 to 0.66, *p* < 0.001, GRADE = moderate; 2 h: RR = 0.38, 95% CI: 0.26 to 0.56, *p* < 0.00001, GRADE = moderate; 6 h: RR = 0.20, 95% CI: 0.11 to 0.38, *p* < 0.00001, GRADE = moderate). The meta-analysis results present in Figure 3.

#### Any Severity Postoperative CRBD

**Figure 4:**
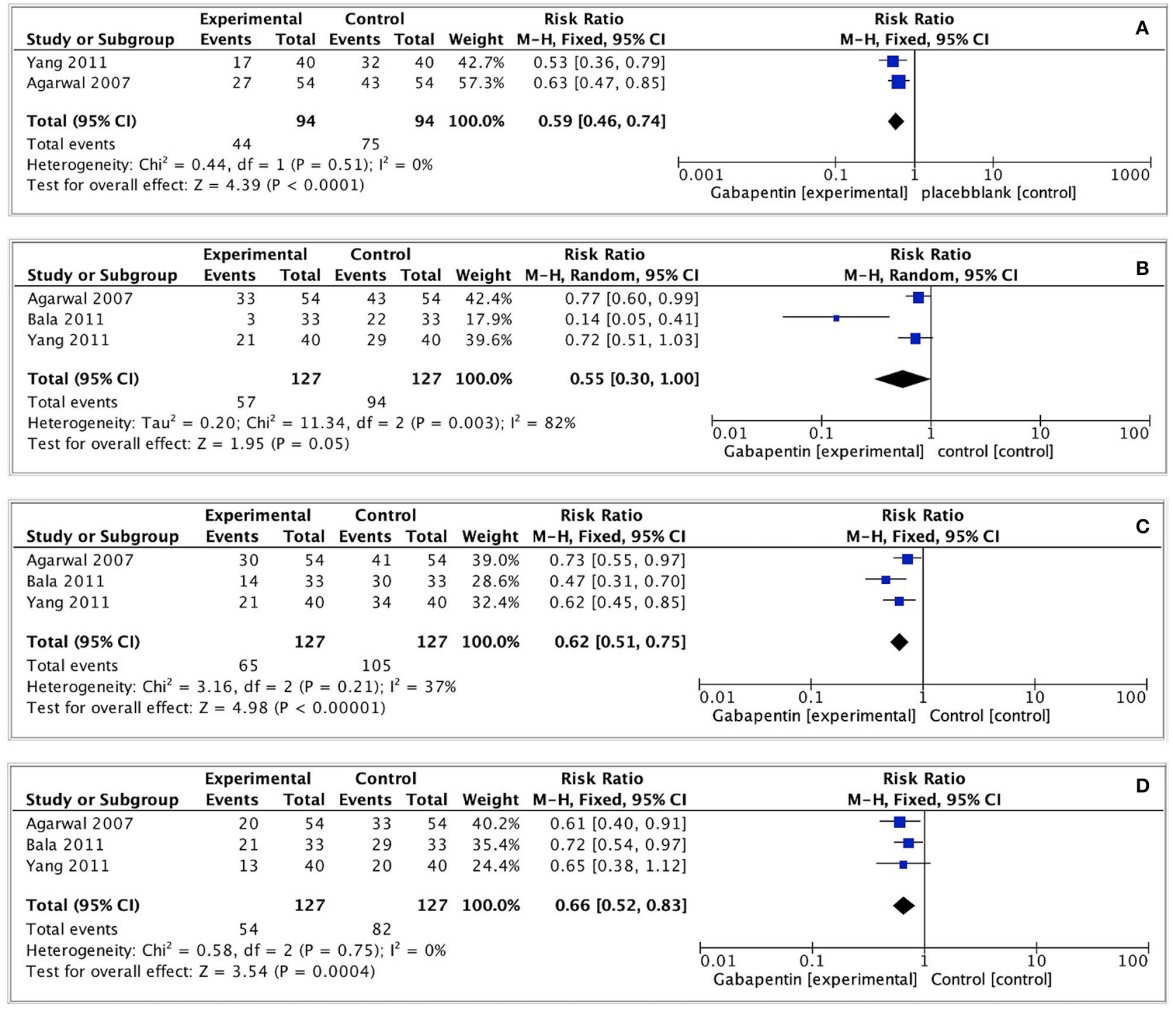
Forest plot of any severity postoperative CRBD. **(A)** Any severity postoperative CRBD at 0h after surgery; **(B)** Any severity postoperative CRBD at 1h after surgery; **(C)** Any severity postoperative CRBD at 2h after surgery; **(D)** Any severity postoperative CRBD at 6h after surgery.

We evaluated the incidence of any grade CRBD at 0, 1, 2, and 6 h after surgery. The pooled effect size was calculated at 0, 2, and 6h (I^2^ = 0%; 37%; 0%) using a fixed effects model, and at 1 h (I^2^ = 82%) using a random effects model. The incidence of CRBD at 1 h was not significantly different between the two groups (RR = 0.55, 95% CI: 0.30 to 1.00, p = 0.05, GRADE = low). Furthermore, the incidence of CRBD was significantly reduced at 0, 2, and 6 h after surgery in the gabapentin group (0 h: RR = 0.59, 95% CI: 0.46 to 0.74, *p* < 0.0001, GRADE = low; 2 h: RR = 0.62, 95% CI: 0.51 to 0.75, *p* < 0.00001, GRADE = moderate; 6 h: RR = 0.66, 95% CI: 0.52 to 0.83, *p* < 0.001, GRADE = moderate). Figure 4 illustrates these results.

#### Postoperative Pain

**Figure 5:**
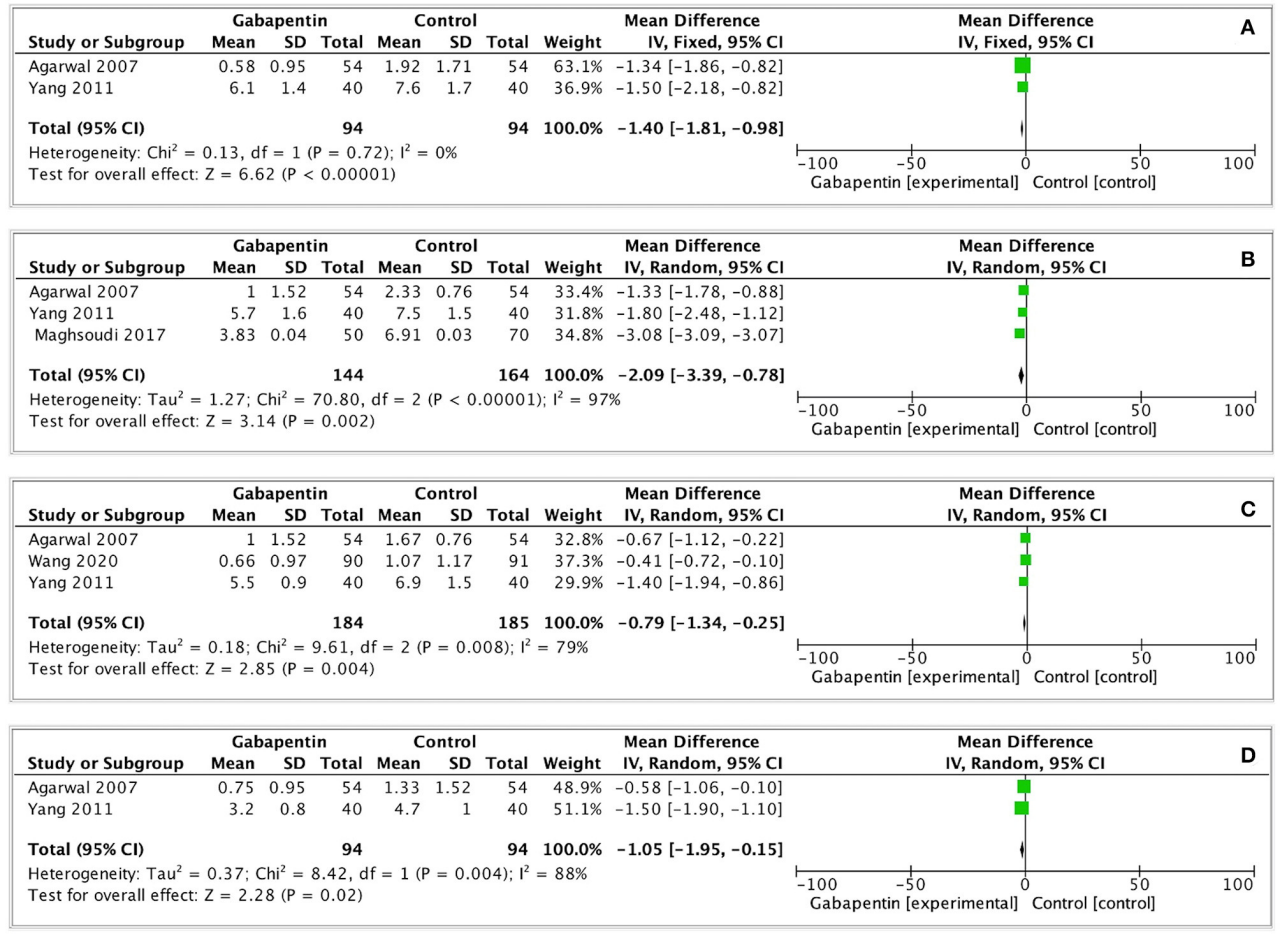
Forest plot of postoperative pain scores. **(A)** Postoperative pain scores at 0h after surgery; **(B)** Postoperative pain scores at 1h after surgery; **(C)** Postoperative pain scores at 2h after surgery; **(D)** Postoperative pain scores at 6h after surgery.

Four of the included studies recorded postoperative pain scores (VAS 0–10) at each time point (15, 17, 18, 20). Slightly low postoperative pain scores were observed in gabapentin group (0 h: MD = −1.40, 95% CI: −1.81 to −0.98, *p* < 0.00001, GRADE = low; 1 h: MD = −2.09, 95% CI: −3.39 to−0.78, *p* = 0.002, GRADE = low; 2 h: MD = −0.79, 95% CI: −1.34 to−0.25, *p* = 0.004, GRADE = low; 6 h: MD = −1.05, 95% CI: −1.95 to−0.15, *p* = 0.02, GRADE = low). The observed statistical heterogeneity (I^2^ = 0%; 97%; 79%; 88%) was not influenced by the type of surgery. The results are shown in Figure 5.

Rescue analgesics was performed in four of the included studies (13, 15, 16, 18), but due to large clinical heterogeneity, pooling of these data and meta-analysis was considered inappropriate. Therefore, a descriptive analysis has been adopted. Two studies used fentanyl as a postoperative analgesic (13, 18). Agarwal et al. (13) reported that gabapentin could significantly reduce total fentanyl administration and the number of patients requiring it postoperatively (*p* < 0.05). The study of Yang et al. (18) reported a decrease in the times of pressing patient-controlled analgesia (PCA) which was filled with fentanyl at 1, 2, and 6 h after surgery (*p* < 0.05). Maghsoudi et al. (15) administered patients with both paracetamol and pethidine (25 mg) to manage postoperative pain. They found that the total consumption of paracetamol and pethidine for 24 h after surgery were significantly lower in the gabapentin group compared with the control group (*p* < 0.001). Wang et al. (16) determined that gabapentin was effective for decreasing postoperative tramadol use. Not only the percentage of patients requiring tramadol and dose of tramadol use had a decrease, but also the time to the first tramadol request had prolonged within 48 h following surgery (*p* < 0.05). The details about postoperative treatment were shown in Supplementary Material.

#### Adverse Events

**Figure 6:**
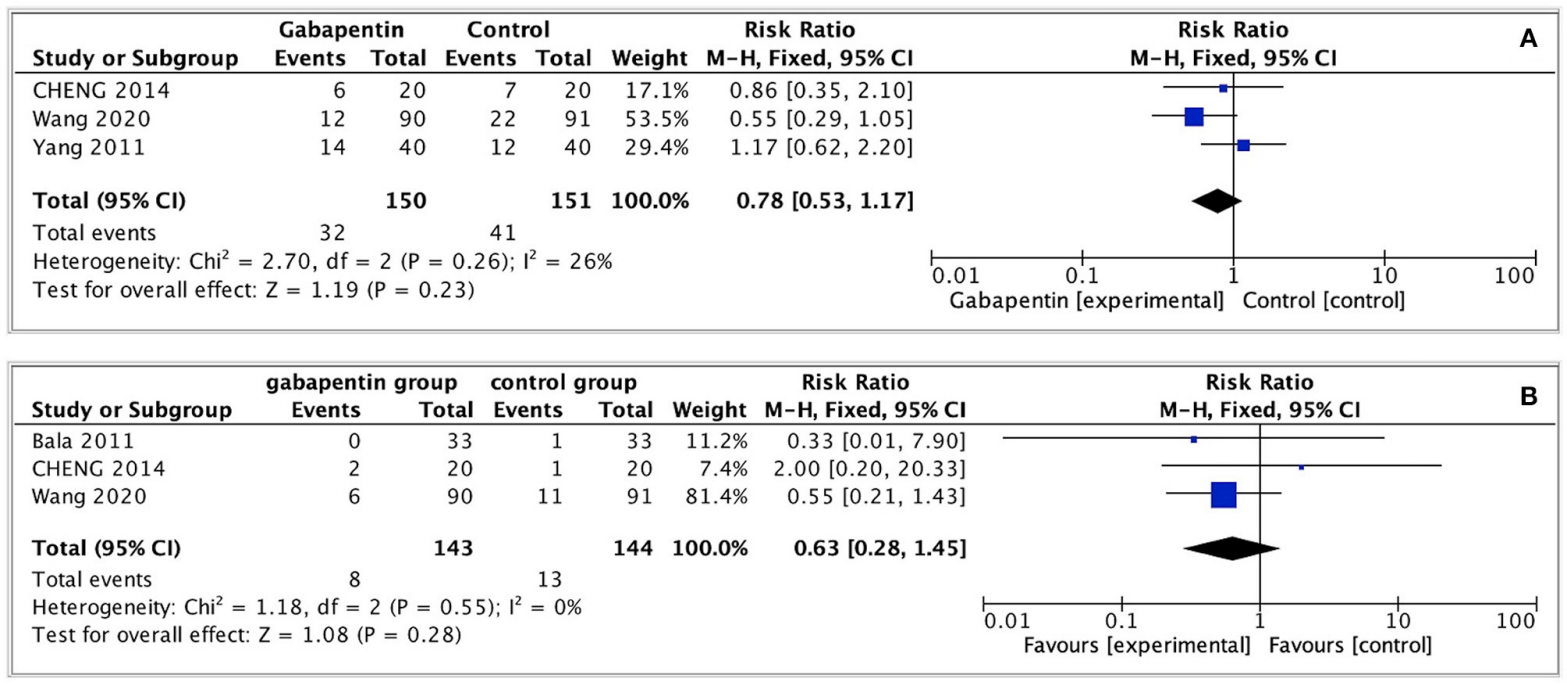
Forest plot of adverse events. **(A)** Nausea and vomiting after surgery; **(B)** Dizziness after surgery.

In previous researches, gabapentin was found associated with the higher risk of side-effects (e.g., dizziness, drowsiness, nausea and vomiting) (19, 20). However, these adverse events of gabapentin are difficult to be distinguished from common postoperative complications causing by some anesthetic drugs. In our analyses, one study (13) reported that no significant difference in postoperative sedation, nausea and vomiting, feeling of light-headedness, or headache between gabapentin and control group (detailed data not shown). One study (15) reported no significant adverse effects were seen during the whole trial. Three studies (16–18) shown that preoperative use of gabapentin was associated with low incidence of postoperative nausea and vomiting; however, no statistically significant differences were observed in postoperative nausea and vomiting between gabapentin and control groups (RR = 0.78, 95% CI: 0.53 to 1.17, *p* = 0.23, I^2^ = 26%, GRADE = moderate). Similarly, no statistically significant difference was observed in the incidence of dizziness (RR = 0.63, 95% CI: 0.28 to 1.45, *p* = 0.28, I^2^ = 0%, GRADE = moderate) (Figure 6).

### Subgroup Analysis

**Figure 7:**
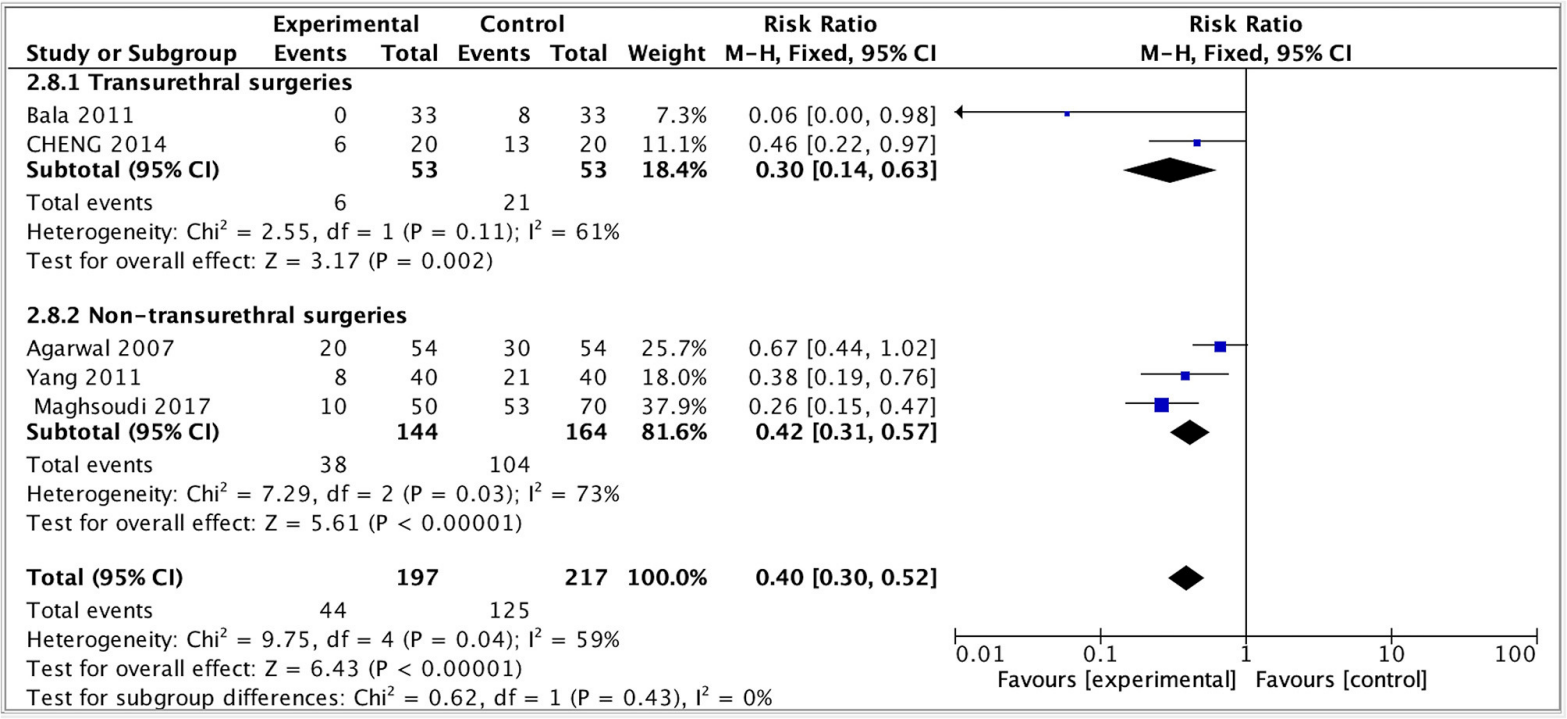
Subgroup analysis.

Given that the different surgery types were crucial variables for the effect evaluation, a subgroup analysis of primary outcome was performed by dividing the study into two subgroups: transurethral and non-transurethral surgery. The result showed that in both groups, the incidence of moderate-to-severe CRBD reduced but the heterogeneity remained high (transurethral: RR = 0.33, 95% CI: 0.14 to 0.63, *p* < 0.01, I^2^ = 61; non-transurethral: RR = 0.42, 95% CI: 0.31 to 0.57, *p* < 0.00001, I^2^ = 73), indicating that whether or not patients received transurethral surgeries was not the main source of heterogeneity and our results were robust (Figure 7).

### Sensitivity Analysis

**Figure 8:**
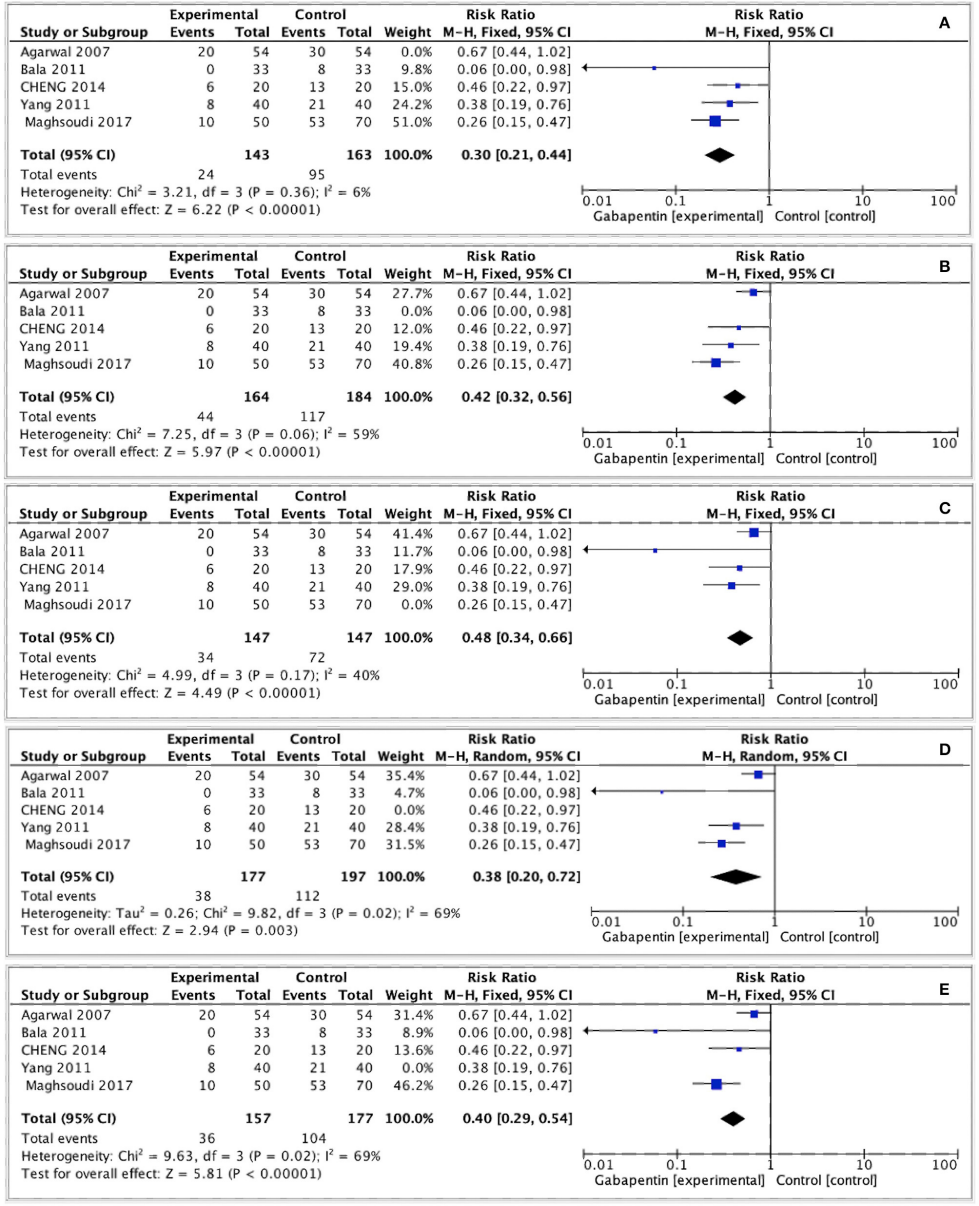
Sensitivity analysis. **(A)** Sensitivity analysis by removing Agarwal's study. **(B)** Sensitivity analysis by removing Bala's study. **(C)** Sensitivity analysis by removing Maghsoudi's study. **(D)** Sensitivity analysis by removing Yang's study. **(E)** Sensitivity analysis by removing Cheng's study.

We carried out sensitivity analyses of the primary outcomes by removing one study at a time. As a result, the pooled outcomes on the moderate-to-severe postoperative CRBD at 1 h after surgery were altered after omitting Agarwal's (13) and Maghsoudi's (15) studies. The other outcomes did not change (Figure 8).

## Discussion

In this systematic review we provide a moderate-to-low level of certainty that preoperative oral gabapentin is associated with lower risk of moderate-to-severe CRBD at 0, 1, 2 and 6 h after surgery. Besides, preoperative oral gabapentin can reduce the overall incidence of CRBD at 0, 2 and 6 h. The findings suggest that gabapentin could be a potential preventive intervention for short-term postoperative CRBD in patients with transurethral catheterization. Although no meta-analysis has been previously conducted on the effects of gabapentin on postoperative CRBD, our results are consistent with previous reviews, which revealed that gabapentin administration could be an effective option for the prevention of CRBD. Bai and her colleagues reviewed 14 articles and concluded that muscarinic antagonists (e.g., gabapentin) and other agents appeared to reduce the incidence of CRBD compared with placebo (4). However, a meta-analysis could not be performed because only two studies regarding gabapentin were included in the analysis. Furthermore, in a network meta-analysis conducted by Hur and his colleagues, gabapentin was ranked best with regard to the overall incidence of CRBD (9). Nevertheless, only two RCTs on gabapentin included in this network meta-analysis and it did not evaluate postoperative pain and adverse events, the evidence remains underpowered.

A decrease in postoperative pain scores in gabapentin group has been observed in our study, but there might be no clinical significance considering appreciable minimally important variations in pain intensity (2 to 3 of 10) (21). In addition, though descriptive analyses we could draw a conclusion that preoperative oral gabapentin could reduce the use of painkillers after surgery. Actually, for a long time, gabapentin has been used for the treatment of many types of peripheral neuropathic pain including post-herpetic neuralgia (22, 23) and painful diabetic peripheral neuropathy (22, 24), but the mechanisms have been not well understood. Some experiments revealed that gabapentin could affect modulation of pain by central nervous system (25, 26), and neuroimaging studies indicated gabapentin might influence brain function in models of central sensitization and in patients with chronic pain (27).

No significant adverse effects were seen in gabapentin group. In a recent meta-analysis conducted for chronic neuropathic pain, compared with placebo, gabapentin was related to more dizziness (19 vs. 7%; *p* < 0.001) and drowsiness (14 vs. 5%; *p* < 0.001) (28). Nevertheless, the rates of side effects in our study were much lower than in these other studies. This would be owing to larger dose gabapentin being used for treating chronic pain (1,200–3,600 mg/d for 4–12 weeks rather than 600 mg for only once before surgery), suggesting that increased gabapentin dose might expose patients to increased risk of side effects.

To the best of our knowledge, this is the first meta-analysis to evaluate the efficacy and safety of gabapentin in the prevention of CRBD in surgical patients. We followed standardized recommendations, and developed well-defined and strict inclusion and exclusion criteria. We also used the Cochrane Collaboration's tool and GRADE system to evaluate the risk of bias and the overall level of certainty. Furthermore, we assessed clinically relevant outcomes from the perspectives of statistical and clinical significance to translate our findings into clinical practice. We undertook a series of subgroup and sensitivity analyses to explore potential sources of heterogeneity, and found that surgical site rather than the type of surgical procedure and anesthesia was the main source of heterogeneity.

However, the current study had several limitations. First, the small number of included studies is a major limitation. We could only conduct a pilot study whose results should be interpreted with caution. And we could not do further subgroup analysis based on the type of surgery and anesthesia, which might have an impact on patients' perception of bladder discomfort and pain. Second, the present study only evaluated the outcomes of gabapentin at 0, 1, 2, and 6 h, and a long-term effect could not be assessed due to insufficient data. Finally, except for nausea, vomiting and dizziness, the risk of other side effects of gabapentin such as postoperative sedation, respiratory depression, delirium, and postoperative ataxia could not be evaluated because the data was lacking.

One commonly accepted mechanism underlying the occurrence of CRBD is the activation of muscarinic acetylcholine receptors stimulated by the indwelling urethral catheter (8). Accordingly, gabapentin, an antimuscarinic agent, has been believed to be a promising prevention strategy. Our study confirmed that preoperative oral administration gabapentin was able to reduce the risk and severity CRBD as well as postoperative pain without significant side effects. Considering the long-term benefits and other potential side effects of gabapentin, we cannot interpret our results as strong evidence in favor of its off-label use. Larger studies are needed to assess the safety profile of this medication.

## Conclusion

As an adjunct to surgery, preoperative oral gabapentin is effective in decreasing the risk and severity CRBD over a short period after surgery, and it can decrease postoperative pain without significant side effects. Nevertheless, further studies are required in future to assess the effectiveness and safety of gabapentin.

## Data Availability Statement

The datasets presented in this study can be found in online repositories. The names of the repository/repositories and accession number(s) can be found in the article/Supplementary Material.

## Author Contributions

F-MY and Y-FR: conception and design. F-MY: administrative support. Y-TW and CX: provision of study materials or patients and collection and assembly of data. HL and XF: data analysis and interpretation. All authors contributed to the article and approved the submitted version.

## Funding

National Administration of Traditional Chinese Medicine: 2019 Project of building evidence-based practice capacity for TCM (2019XZZX-ZL006); Provincial Developmental Fund of Traditional Chinese Medicine - Key Discipline of TCM (Oncology of TCM) (2100601).

## Conflict of Interest

The authors declare that the research was conducted in the absence of any commercial or financial relationships that could be construed as a potential conflict of interest.

## Publisher's Note

All claims expressed in this article are solely those of the authors and do not necessarily represent those of their affiliated organizations, or those of the publisher, the editors and the reviewers. Any product that may be evaluated in this article, or claim that may be made by its manufacturer, is not guaranteed or endorsed by the publisher.
