# Supplementary material for: Preoperative Oral Gabapentin in the Management of Postoperative Catheter-Related Bladder Discomfort in Adults: A Systematic Review and Meta-Analysis
Source: Front Surg. 2021 Oct 18;8:755497. doi: 10.3389/fsurg.2021.755497 (PMC8558345; doi:10.3389/fsurg.2021.755497)
Supplement: Supplementary file 1 [file Table_1.docx]

**Standard criteria for bladder discomfort**

| **Score** | **Grade** | **Descriptions** |
| --- | --- | --- |
| 0 | no | patient do not complain of any bladder discomfort |
| 1 | mild | patient report bladder discomfort rather than surgical pain only on being questioned |
| 2 | moderate | patient report bladder discomfort without being questioned; not accompanied by any behavioral responses |
| 3 | severe | patient report bladder discomfort without being questioned and accompanied by behavioral responses such as flailing limbs, strong vocal response, or attempts to pull the catheter out |
