# Supplementary material for: Preoperative Oral Gabapentin in the Management of Postoperative Catheter-Related Bladder Discomfort in Adults: A Systematic Review and Meta-Analysis
Source: Front Surg. 2021 Oct 18;8:755497. doi: 10.3389/fsurg.2021.755497 (PMC8558345; doi:10.3389/fsurg.2021.755497)
Supplement: Supplementary file 2 [file Table_2.docx]

| **Study** | **Drug** | **Mode of administration** | **Time after surgery (h)** | **Indicator** | **Outcome** | | |
| --- | --- | --- | --- | --- | --- | --- | --- |
|  |  |  |  |  | **Gabapentin group** | **Control group** | **P value** |
| Agarwal 2007 | Fentanyl | PCA | 6 | Total fentanyl requirement (ug.kg^-1^.h^-1^) | 0.5±0.2 | 0.9±0.3 | ＜0.05 |
| Bala 2012 | Hyoscine butyl bromide | i.v. | - | No record | - | - | - |
| Maghsoudi 2018 | Pethidine | i.m. | 24 | The number of injections(n) | 0.68±0.62 | 2.4±0.64 | ＜0.001 |
|  | Paracetamol | i.m. | 24 | The number of injections (n) | 1.8±0.7 | 3.6±0.7 | ＜0.001 |
| Wang 2020 | Tramadol | i.v. | - | Time to the first tramadol request (min) | 123.8±49.7 | 101.6±41.8 | 0.0014 |
|  |  |  | 48 | Patients requiring tramadol analgesia (%) | 57 (63.3) | 72 (79.1) | 0.029 |
|  |  |  | 48 | Dose of tramadol received (1.5mg·kg^-1^) (n) | 83 | 117 | 0.0387 |
| Yang 2011 | Fentanyl | PCA | 6 | The times of pressing PCA (n) | 20.7±1.2 | 34.2±1.8 | ＜0.05 |
| Cheng 2014 | No | - | - | - | - | - | - |

**Postoperative drug treatments**

PCA：patient-controlled analgesia

i.v.: Intravenous injection

i.m.: intramuscular injection
