# Supplementary material for: Preoperative Oral Gabapentin in the Management of Postoperative Catheter-Related Bladder Discomfort in Adults: A Systematic Review and Meta-Analysis
Source: Front Surg. 2021 Oct 18;8:755497. doi: 10.3389/fsurg.2021.755497 (PMC8558345; doi:10.3389/fsurg.2021.755497)
Supplement: Supplementary file 3 [file Data_Sheet_1.pdf]

Search strategies

Table 1. The search strategies and results of PubMed

Number	Search terms	Number
#1	(((((((Gabapentin[MeSH Terms]) OR (Gabapentin*[Title/Abstract])) OR (1-(Aminomethyl)cyclohexaneacetic[Title/Abstract])) OR (Neurontin[Title/Abstract])) OR (Convalis[Title/Abstract])) OR (Novo-Gabapentin[Title/Abstract])) OR (Novo Gabapentin[Title/Abstract])) OR (NovoGabapentin[Title/Abstract])	7463
#2	(((Catheter-related bladder discomfort) OR (Catheter related bladder discomfort)) OR (bladder discomfort)) OR (bladder)	192633
#3	(((Urinary Catheterization[MeSH Terms]) OR (Catheterization[MeSH Terms])) OR (Catheterization*)) OR (Cannulation*)	238763
#4	#1 and (#2 OR #3)	70

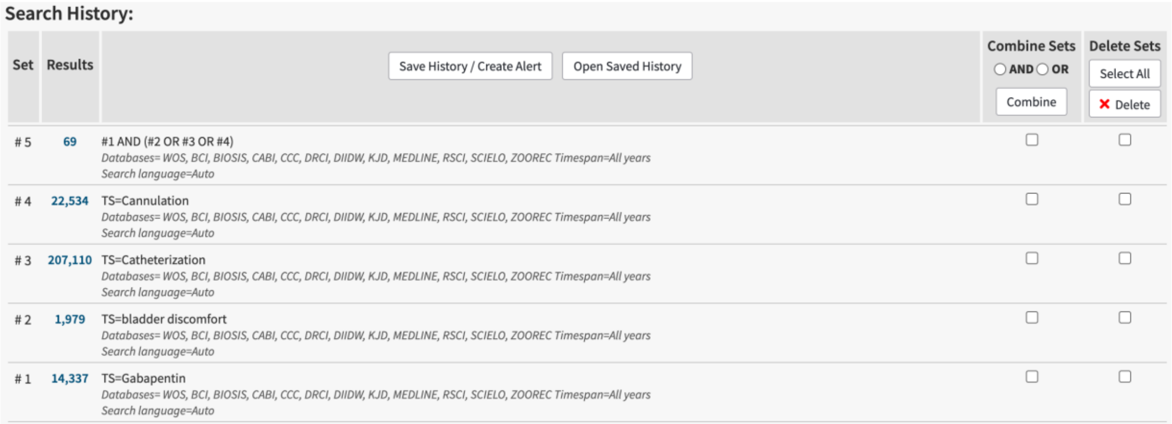

Search History:

Set	Results	Save History / Create Alert · Open Saved History	Combine Sets (AND / OR, Combine)	Delete Sets (Select All, Delete)
# 5	69	#1 AND (#2 OR #3 OR #4)<br>*Databases= WOS, BCI, BIOSIS, CABI, CCC, DRCI, DIIDW, KJD, MEDLINE, RSCI, SCIELO, ZOOREC Timespan=All years*<br>*Search language=Auto*		
# 4	22,534	TS=Cannulation<br>*Databases= WOS, BCI, BIOSIS, CABI, CCC, DRCI, DIIDW, KJD, MEDLINE, RSCI, SCIELO, ZOOREC Timespan=All years*<br>*Search language=Auto*		
# 3	207,110	TS=Catheterization<br>*Databases= WOS, BCI, BIOSIS, CABI, CCC, DRCI, DIIDW, KJD, MEDLINE, RSCI, SCIELO, ZOOREC Timespan=All years*<br>*Search language=Auto*		
# 2	1,979	TS=bladder discomfort<br>*Databases= WOS, BCI, BIOSIS, CABI, CCC, DRCI, DIIDW, KJD, MEDLINE, RSCI, SCIELO, ZOOREC Timespan=All years*<br>*Search language=Auto*		
# 1	14,337	TS=Gabapentin<br>*Databases= WOS, BCI, BIOSIS, CABI, CCC, DRCI, DIIDW, KJD, MEDLINE, RSCI, SCIELO, ZOOREC Timespan=All years*<br>*Search language=Auto*		

Figure 1. The search strategies and results of Web of Science

History Save | Delete | Print view | Export | Email Combine using And Or Collapse

#5	#1 AND (#2 OR #3 OR #4)	38
#4	**cannulation**:ab,ti	21,554
#3	**catheterization**:ab,ti	79,134
#2	**bladder** AND **discomfort**:ab,ti	2,057
#1	**gabapentin**:ab,ti	10,309

Figure 2. The search strategies and results of Embase

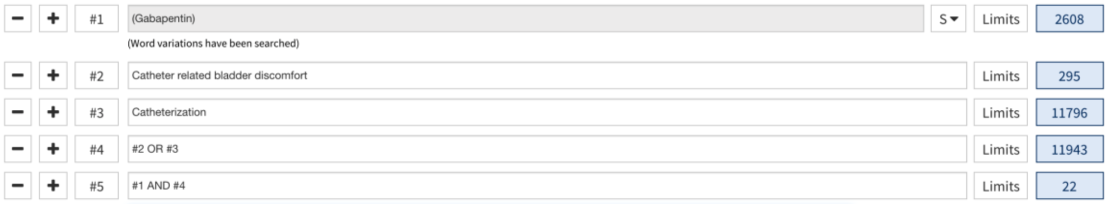

#1	(Gabapentin) (Word variations have been searched)	2608
#2	Catheter related bladder discomfort	295
#3	Catheterization	11796
#4	#2 OR #3	11943
#5	#1 AND #4	22

Figure 3. The search strategies and results of Cochrane Library

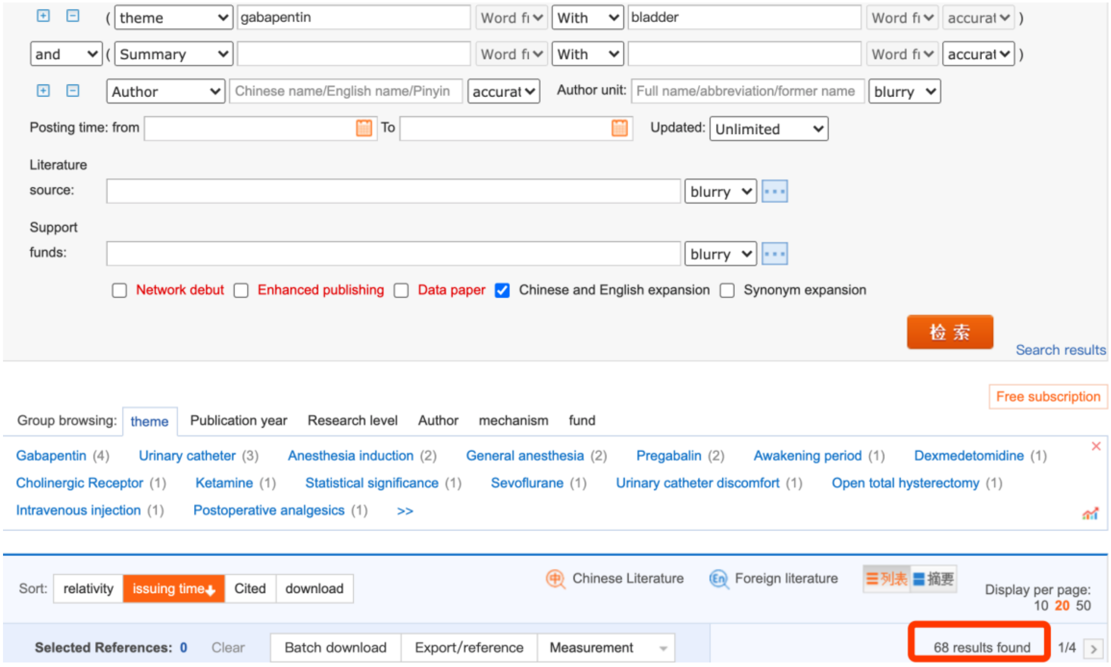

Figure 4. The search strategies and results of CNKI

Search history

Current search history | Collection search history

Search	Search Results	Retrieval time	Add to search query	operating
#1, (gabapentin) AND bladder	67	2021/02/06	AND OR	Favorites

Figure 5. The search strategies and results of Wan Fang
